# The MAMs Structure and Its Role in Cell Death

**DOI:** 10.3390/cells10030657

**Published:** 2021-03-16

**Authors:** Nan Wang, Chong Wang, Hongyang Zhao, Yichun He, Beiwu Lan, Liankun Sun, Yufei Gao

**Affiliations:** 1 China Japan Union Hospital, Jilin University, Changchun 130031, China; wnan18@mails.jlu.edu.cn (N.W.); chongwang19@mails.jlu.edu.cn (C.W.); zhaohy18@mails.jlu.edu.cn (H.Z.); heyc17@mails.jlu.edu.cn (Y.H.); lanbw20@mails.jlu.edu.cn (B.L.); 2 Key Laboratory of Pathobiology, Ministry of Education, Department of Pathophysiology, College of Basic Medical Sciences, Jilin University, Changchun 130012, China

**Keywords:** endoplasmic reticulum, mitochondria, MAMs, Ca^2+^, apoptosis

## Abstract

The maintenance of cellular homeostasis involves the participation of multiple organelles. These organelles are associated in space and time, and either cooperate or antagonize each other with regards to cell function. Crosstalk between organelles has become a significant topic in research over recent decades. We believe that signal transduction between organelles, especially the endoplasmic reticulum (ER) and mitochondria, is a factor that can influence the cell fate. As the cellular center for protein folding and modification, the endoplasmic reticulum can influence a range of physiological processes by regulating the quantity and quality of proteins. Mitochondria, as the cellular “energy factory,” are also involved in cell death processes. Some researchers regard the ER as the sensor of cellular stress and the mitochondria as an important actuator of the stress response. The scientific community now believe that bidirectional communication between the ER and the mitochondria can influence cell death. Recent studies revealed that the death signals can shuttle between the two organelles. Mitochondria-associated membranes (MAMs) play a vital role in the complex crosstalk between the ER and mitochondria. MAMs are known to play an important role in lipid synthesis, the regulation of Ca^2+^ homeostasis, the coordination of ER-mitochondrial function, and the transduction of death signals between the ER and the mitochondria. Clarifying the structure and function of MAMs will provide new concepts for studying the pathological mechanisms associated with neurodegenerative diseases, aging, and cancers. Here, we review the recent studies of the structure and function of MAMs and its roles involved in cell death, especially in apoptosis.

## 1. Introduction

Apoptosis is a programmed form of cell death and can occur via three main pathways. The first pathway is the intrinsic apoptosis pathway; initiated by a range of factors, including the effects of growth factors or hormones, radiation, or cytotoxins [1]. This process involves the enhancement of pro-apoptotic signals and the weakening of anti-apoptotic signals. An imbalance in the regulation of apoptosis ultimately leads to changes in the permeability of the mitochondrial outer membrane and the release of pro-apoptotic substances from the mitochondria. These pro-apoptotic substances promote apoptosis by activating the apoptotic executive protein caspase-9, inhibiting IAPs or via the direct cleavage of DNA [2,3,4,5,6,7]. The second pathway is the extrinsic apoptosis pathway in which apoptosis is activated by the binding of specific ligands such as FasL to transmembrane receptors which contain “death domain” such as FasR. Activated death receptors then recruit adaptor proteins in the cytoplasm to assemble an apoptosis-inducing signal complex, which then activates caspase-8 to initiate apoptosis [8]. Caspase-8 can also cleave Bid to initiate the intrinsic apoptotic pathway, which plays an important role in the process of apoptotic signal amplification [9,10]. The third pathway is the perforin/granzyme pathway in which cytotoxic T cells or NK cells induce target cell apoptosis by secreting granules containing perforin or granzymes [11].

Moreover, the Bcl2 family plays a vital role in cell apoptosis. The Bcl2 family consists of 25 members, and they can be divided into pro-apoptotic proteins (Bax, Bak, etc.) and anti-apoptotic proteins (Bcl2, Bcl-XL, Mcl-1, etc.) according to their different functions. Furthermore, pro-apoptotic proteins can be divided into pro-apoptotic proteins with multiple domains and BH3-only proteins, and BH3-only proteins also can be divided into “activator” (Bim, tBid, etc.) and “sensitizer” (Bad, Bik, etc.) based on their specific mechanism of action [12]. The activated pro-apoptotic members can assemble on the outer mitochondrial membrane and change the permeability of the outer mitochondrial membrane, and promote the release of cytochrome c, AIF, Smac/Diablo and other apoptosis-inducing factors from the mitochondria [13]. Anti-apoptotic members mainly antagonize the effects of pro-apoptotic ones through protein–protein interactions to maintain the integrity of the mitochondrial outer membrane. The “activator” BH3-only members can directly activate the pro-apoptotic proteins and promote the occurrence of apoptosis, while the “sensitizer” BH3-only members can interact with the anti-apoptotic in a protein–protein interaction way to relieve the effect of the pro-apoptotic proteins [14].

As it is mentioned above, the mitochondria play a central role in the cell apoptosis, and the crosstalk between mitochondria and other organelles may impact the apoptosis process. Recent studies revealed that the communication between ER and mitochondria can influence the cell apoptosis, thus affecting the cell fate.

The ER is a key organelle that plays a crucial role in Ca^2+^ storage, lipid synthesis, protein folding, and assembly [15,16,17,18]. Mitochondria are the “energy factories” of eukaryotic cells, and provide energy to drive the physiological processes of cells; they also play a key role in the process of apoptosis [19,20,21]. The ER and mitochondria are independent of each other but are also closely associated in structure and function. The first spatial connection between the ER and the mitochondria was reported in the 1950s following a study of hepatocytes by transmission electron microscopy [22,23]. In 2006, an electron tomography study further confirmed the complex relationship between the ER and the mitochondria [24]. It is now believed that the mitochondrial surface juxtaposed to the ER in mammalian cells is up to 5–20% due to different cell types [25,26]. Based on this close structural connection, the ER is able to respond to a variety of stress stimuli and can transmit these stress signals to the mitochondria [27,28], thereby initiating the mitochondrial stress response. Similarly, the mitochondria can transmit signals to the ER, thus ensuring the efficient execution of compensatory responses or cell death events. Due to the special function of these precise structural associations, this biological system is usually investigated as a relatively independent sub-organelle structure referred to as a “mitochondrial-related membrane structure.”

## 2. The Structural Characteristics of MAMs

The structure of MAMs does not remain constant; rather, the structure of MAMs changes dynamically in response to the cell status. The width of the gap between the ER and the outer mitochondrial membrane varies from 10 to 100 nm [29,30]; the width of this gap is usually 10–15 nm at the smooth endoplasmic reticulum and 20–30 nm at the rough endoplasmic reticulum; these spatial differences may be related to the presence of ribosomes [7,31]. Different proteomic analysis of the structure of MAMs has revealed 991 [32] and 1212 [33] different proteins in MAMs [34]. Mass spectrometry analysis divided these constituent proteins into three categories: Proteins that are specifically present in MAMs; proteins that exist simultaneously in MAMs and other organelle structures; and proteins that only exist temporarily in MAMs [33]. These proteins are involved in a wide range of processes, such as structural maintenance, lipid synthesis, the regulation of Ca^2+^ homeostasis, mitochondrial dynamics, and apoptosis.

## 3. The Structure Maintenance of MAMs

**Figure 1:**
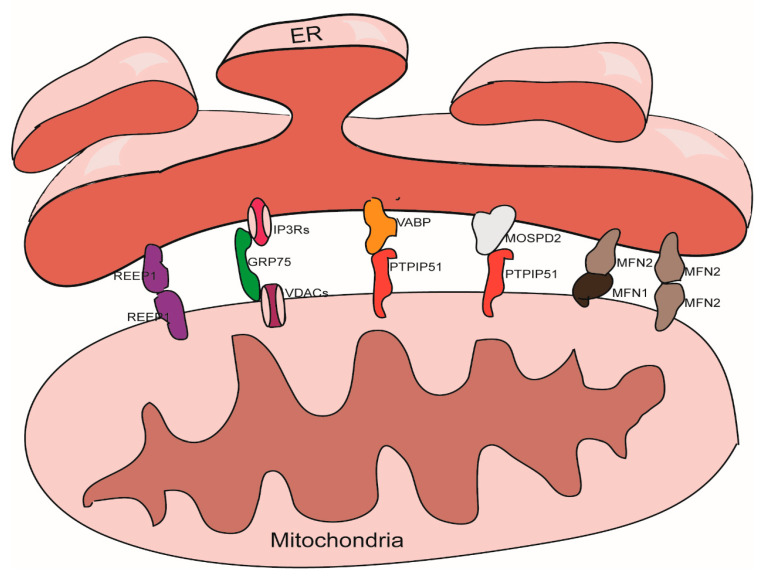
Tethering proteins that participate in the mitochondria-associated membranes (MAMs) structure maintenance.

There are thousands of proteins in MAMs; the roles of these proteins are known to vary widely. Some of these proteins play a tethering role in the maintenance of MAMs [31]. According to our understanding, tethering proteins should exhibit certain characteristics. For example, tethering proteins could be (1) proteins or protein complexes that directly participate in the physical connection between the ER and the mitochondria, or (2) interfering proteins or protein complexes that can directly cause changes in the width of gap, the area of contact, or the number of contact sites between the ER and the mitochondria. These proteins and protein complexes are introduced below (Figure 1).

### 3.1. The IP3Rs-Grp75-VDACs Complex

IP3Rs are important Ca^2+^ outflow channels on the surface of the ER and mediate the release of Ca^2+^ from the cavity of the ER to the cytoplasm [35,36]. VDACs are ions channel located on the outer membrane of the mitochondria; these mediate the movement of a variety of ions and metabolites in and out of mitochondria, and participate in a range of cellular activities, including apoptosis, metabolism, and the regulation of Ca^2+^ [37]. IP3Rs and VDACs are connected by Grp75 to maintain the structure of MAMs [38]. The overexpression of VDACs is known to enhance the connection between the ER and the mitochondria and thus improve Ca^2+^ flux from the ER to the mitochondria [39]; while silencing VDAC1 exhibits a reduction in the connection between Grp75 and IP3R1 indicating a reduction of ER-mitochondria interactions [40]. Cells overexpressing Grp75 showed higher number of IP3R1–VDAC1 interaction sites [41]. Silencing IP3R1 or Grp75 can also reduce the connection between VDAC1 and Grp75 or IP3R1 [42]. 

### 3.2. The VAPB-PTPIP51 Complex

VAPB is located in the membrane of the ER and participates to the activation of the IRE1/XBP1 axis in the ER unfolded protein response [43,44]. VAPB can form a complex with the outer mitochondrial membrane protein PTPIP51 and help to maintain the structure of MAMs. A mutant form of VAPB, VAPBP56S, exhibits a stronger affinity for PTPIP51, thereby promoting the transfer of Ca^2+^ from the ER to the mitochondria; knocking out either of these two genes can reduce the transfer of Ca^2+^ signals [45]. Other studies have shown that knocking down either of these two proteins will reduce the level of contacts between the ER and the mitochondria [46,47]. 

### 3.3. The Mfn1/Mfn2 Complex

In addition to being located in the outer mitochondria membrane and participating to the mitochondrial fusion [48], Mfn2 can also localize on the surface of the ER. Mfn2 participates in the structural maintenance of MAMs by forming homodimers or heterodimers with Mfn1/2 on the outer membrane of the mitochondria. The function of the Mfn1/Mfn2 complex with regards to maintaining the structure of the MAMs was first discovered in 2008 [49]; this role has also been confirmed by several other studies [50,51]. However, some studies have yielded contradictory results [52,53], it is now well established that Mfn2 plays a role in the endoplasmic reticulum stress (ERS) response; the ERS induced by knockdown of Mfn2 can tighten the association between the ER and the mitochondria [54]. 

### 3.4. The MOSPD2-PTPIP51 Complex

MOSPD2, another member of VAP family, a protein that locates on the surface of the ER membrane, plays a role in connecting the ER with other membrane structures. It can also bind with proteins containing a small VAP-interacting motif, named FFAT [two phenylalanines (FF) in an acidic track (AT)] via an MSP (Major Sperm Protein domain), such as PTPIP51 on the outer membrane of the mitochondria [55]. 

### 3.5. REEP1

REEP1 is a protein that is located in the outer membrane of the ER and the mitochondria. REEP1 helps regulate the morphology of the ER. Studies have shown that REEP1 directly connects the ER and the mitochondria through oligomerization and participates in forming the structure of MAMs. In addition, through bending ER membranes, REEP1 makes it topologically possible for the ER to wrap around the mitochondria, which helps to form MAMs [56].

### 3.6. Other Proteins Involved in MAMs Maintance

In addition to these tethering proteins, there are some proteins that do not directly participate in the structural maintenance of MAMs. However, these proteins do affect the structure of MAMs via protein–protein interactions (Table 1). In addition to being present in the cytoplasm, α-Synuclein can also be incorporated in MAMs [57]. α-Synuclein can promote the Ca^2+^ transfer from ER to mitochondria by increasing the ER and mitochondria contacts; and further study showed that the C-terminal of α-Synuclein is essential to tighten the contacts [58]. Some studies revealed that the α-Synuclein existing in MAMs results in the dis-regulation of Ca^2+^ and lipid metabolism, which promotes substantia nigra pars compacta neurons to die, leading to the progression of PD [59]. In addition to playing an anti-apoptotic role in cells and participating in mitochondrial dynamics, DJ-1 can still exist in the MAMs, thus enhancing the connection between the ER and the mitochondria and the crosstalk between the two organelles; this effect may be related to P53 to some extent (an antagonistic relationship) [60]. Existing studies suggest that DJ-1 can bind directly to the IP3R-Grp75-VDAC complex and affect its stability. The knockout of DJ-1 resulted in the aggregation of IP3R3 in MAMs and a reduction in the formation of the IP3Rs-Grp75-VDACs complex; it is possible that this is related to the pathophysiological process of obesity [61]. Although the precise mechanism remains obscure, it has been ascertained that DJ-1 can affect the structural stability of MAMs. This also implies that MAMs may play a role in the pathogenesis of Parkinson’s syndrome. TDP-43 and FUS are proteins that are related to ALS/FTD and can activate GSK-3b by down-regulating the phosphorylation levels of serine 9 by GSK-3b. Once activated, GSK-3b can reduce the connections between VABP and PTPIP51, thereby detaching the ER from the mitochondria [47,62]. PDK4 can directly interact with the IP3Rs-Grp75-VDACs complex in MAMs and may promote the formation of this complex by regulating phosphorylation, thus increasing the area of contacts between the ER and the mitochondria [42]. In addition to participating in the post-transcriptional modification of proteins, TG2 can also be incorporated in MAMs and act directly on Grp75 to increase the number of ER-mitochondrial contacts and thus participate in the structural maintenance of MAMs [63]. The precise function of TpMs (a type of keratin binding protein that is partly located in the mitochondria) remains unclear although data indicates that this protein can negatively regulate the ER-mitochondria connections in a Mfn2-dependent manner [64]. It is generally believed that CypD, a protein located in the mitochondrial matrix, can also be incorporated in MAMs, and directly act with the IP3Rs-Grp75-VDACs complex to regulate the stability of this complex. Inhibiting the function of CypD can down-regulate the binding of Grp75 with IP3Rs and VDACs, affecting the transfer of Ca^2+^ between the two organelles [65]. FUNDC1 is known for maintaining the stability of IP3R2 in MAMs by direct binding, and it enhances the level of contacts and the communication of Ca^2+^ between the ER and the mitochondria [66]. Presenilin-2 can also promote the connection and the transfer of Ca^2+^ signals between the ER and the mitochondria in the presence of Mfn-2; these findings were confirmed by overexpression and knockdown experiments, which suggested that presenilin-2 works with the Mfn1/Mfn2 complex [67]. FATE1 can reduce the level of contacts between the ER and the mitochondria and downregulate the transfer of Ca^2+^ with an impaired sensitivity to Ca^2+^-related apoptosis [68]. In addition to participating in the morphological regulation of ER, NogoB can increase the gap width of MAMs and affect their function [69]. PERK, which plays an important role in ERS, can increase the level of connectivity between the ER and the mitochondria by interacting with Mfn2, and thus promote the transduction of ERS signals to the mitochondria [70,71]. Although these proteins are not considered to be directly involved in maintaining the structure of MAMs, they still attract research attention due to their specific regulatory effects on the structure of MAMs and their involvement in the pathological processes underlying many neurodegenerative diseases.

## 4. MAMs and Cell Death

**Figure 2:**
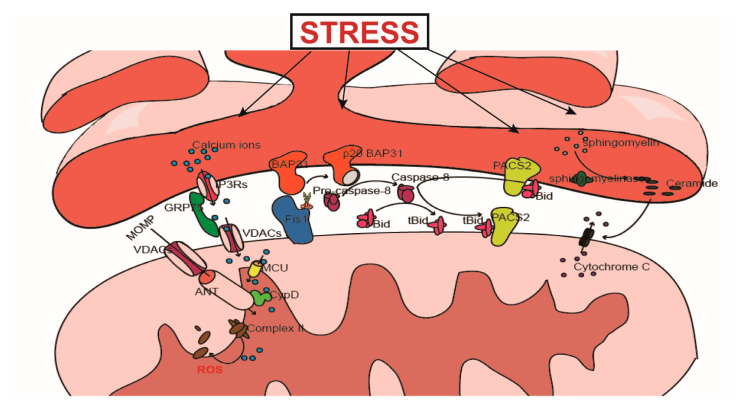
Under stress conditions, endoplasmic reticulum (ER) can act as a sensor and respond immediately. Meanwhile, if the stress intensity exceeds the adaptability of the ER, it will transmit death signals to mitochondria to initiate death events. The transduction can go through some pathways as follows: 1.ER perfuse Ca^2+^ into mitochondria and massive Ca^2+^ in mitochondria works as a death signal initiating cell death; 2. translocation of PACS1 from ER to mitochondria, which is along with the activation and translocation bid; 3. ceramide synthesis and accumulation causing the mitochondrial outer membrane permeabilization (MOMP), which results in the release of cytochrome c or other pro-apoptotic substances in inter membranes space. Also, as a molecule downstream of mitochondrial apoptosis, Fis1 can transmit apoptosis signal back to ER by cleaving BAP31. The ER-mitochondria-ER amplification loop of apoptotic signals can help to coordinate the death events between the two organelles.

MAMs plays a crucial role in cell homeostasis as mentioned above, besides, recent studies revealed that MAMs can also influence the cell death events. The death signals in MAMs attend in multiple forms, such as the transfer of Ca^2+^ from the ER to the mitochondria, regulation of protein–protein interactions, the translocation of molecules or the control of lipid metabolism. These processes are described in detail in the following sections (Figure 2).

### 4.1. Ca^2+^-Mediated Signal Transduction and Cell Death

#### 4.1.1. The Physiological Role of Ca^2+^

Ca^2+^ is an important vector for crosstalk between the ER and the mitochondria [72]. The ER, as the most important cellular reservoir of Ca^2+^, maintains proper Ca^2+^ level by Ca^2+^ pumps SERCAs [73]. SERCAs have varied isoforms, among which the ubiquitous SERCA2b shows the highest affinity to Ca^2+^ uptake from the cytoplasm [26]. ER releases Ca^2+^ into the cytoplasm via RyRs and IP3Rs. The IP3Rs are more ubiquitously expressed while RyRs mostly expressed in skeletal muscle, heart and brain [74]. IP3Rs have three isoforms and all of them can be activated by IP3, Ca^2+^, Ca^2+^-binding proteins, ATP, thiol modification and phosphorylation [75]. Although cytoplasmic Ca^2+^ can almost pass freely through the VDACs of the outer mitochondrial membrane, the MCU located in the inner mitochondrial membrane has a weak affinity for Ca^2+^ (Kd 15–20 µM), meanwhile, the concentration of Ca^2+^ in the cytoplasm fluctuates between 50 and 100 nM [16,76]. The mechanism responsible for the uptake of cytoplasmic Ca^2+^ by the mitochondria has been debated by the academic community for many years. Many models, such as Ca^2+^ microdomain hypothesis, was proposed to explain the Ca^2+^ uptake mechanism [25,77,78]. However, with the deepen understand of the MAMs, especially the IP3Rs-Grp75-VDACs complex, advancement was made with this respect. When the ER calcium channels are opened, a hotspot of calcium will be produced in microdomain between the ER and the mitochondria; this makes it possible for the mitochondria to uptake Ca^2+^ [16]. In this process, the IP3Rs-Grp75-VDACs complex plays a crucial role, though there exist some proteins, such as the MiCU family, MCUR1 and SLC25A23, play regulatory role in the work of MCU, which may affect the Ca^2+^ uptake efficiency, and the elegant mechanism can be seen in the review by Belosludtsev KN et al. [79]. 

The presence of Ca^2+^ in the mitochondrial matrix can play a variety of roles. First, it can promote the efficiency of the tricarboxylic acid cycle and the electron transport chain (ETC). Ca^2+^ is known to increase the activities of pyruvate dehydrogenase, ketoglutarate dehydrogenase, and isocitrate dehydrogenase, in the tricarboxylic acid cycle [30] and can directly stimulate ETC-related complexes, thus improving the activity of ATP synthase and promoting the production of ATP [80,81]. It has also been reported that Ca^2+^ can affect metabolism by regulating the activity of glucose transporters [82]. Second, long-term and high-level Ca^2+^ overload can induce cell death. Ca^2+^ can induce the opening of mPTP, which may result in necrosis [83], or the resultant mitochondrial swelling and outer membrane rupture in limited MPT (the open of mPTP does not involve the entire mitochondrial network, and it may appear as “flickering” mode in a small portion of mitochondria) can result in the release of pro-apoptotic substances such as cytochrome c and AIF [84,85,86]. An excessive concentration of Ca2+ in the mitochondria can bind to cardiolipin in the inner membrane of mitochondria to promote the disintegration of respiratory chain complex II, thus leading to the release of multiple subunits; this also induces the production of large amounts of ROS, thereby inducing apoptosis [87]. Like a double-edged sword, Ca^2+^ can both promote metabolism and induce cell apoptosis. The flux of Ca^2+^ determines which cellular events occur, thus highlighting the importance of MAMs structure to cells.

#### 4.1.2. The Regulatory Effect of MAMs on Ca^2+^ Transfer

The IP3Rs-Grp75-VDACs complex is the basis for Ca^2+^ regulation in MAMs; the importance of this complex is the maintenance of physical contacts between the ER and the mitochondria, while enables the combination of IP3Rs and VDACs to overcome the MCU’s low affinity for Ca^2+^, thus increasing the sensitivity and efficiency of Ca^2+^ delivery. Also, another MAMs-presented protein, SERCAs, is fundamental to regulate the Ca^2+^ level in ER, whose activity can affect the Ca^2+^ flux pass through MAMs. 

##### The IP3Rs-Grp75-VDACs Complex

A variety of proteins regulate the transfer of Ca^2+^ between the ER and the mitochondria by interacting with the IP3Rs-Grp75-VDACs complex. mTORC2 can accumulate in MAMs under the stimulation of various growth factors; and mTORC2 in MAMs can phosphorylate Akt and elevate its activity. Moreover, activated Akt can elevate the phosphorylation level of IP3Rs, thus reducing the release of Ca^2+^ in the ER and antagonizing cell apoptosis [88,89,90,91,92]. Antagonistically, PML can affect the phosphorylation level and activity of Akt by elevating the activity of phosphatase PP2A to regulate the function of IP3Rs [93]. As an important protein encoded by tumor suppressor genes, PTEN can counteract Akt and promote Ca^2+^ transfer from the ER to the mitochondria, thus increasing the sensitivity of cells to apoptotic stimuli [94]. 

As an important player that can affect cell fate, the functional role of the Bcl2 family may depend on the interaction between Bcl2s and MAMs, at least to a certain extent. Bcl2 and Bcl-xl can directly bind to the central regulatory domain of IP3Rs or indirectly affect the phosphorylation level of IP3Rs to inhibit the function of IP3Rs; they can also interact with VDAC1 to reduce the uptake of Ca^2+^ by the mitochondria [13,95,96]. In addition to directly inhibiting the activity of IP3Rs [97], Mcl-1, which is also a member of Bcl2 family, can enhance cellular metabolism by interacting with VDACs to promote the transfer of Ca^2+^ [98]. Bok, a pro-apoptotic member of the Bcl2 family, can interact with IP3Rs and change the ratio of IP3R1 and IP3R2 by cleavage; this process is carried out by caspase-3 and results in an increased Ca^2+^ transfer and increased cellular sensitivity to apoptosis [99,100]. More elegant details in this field can be seen in the review by Lewis A, et al. [101]. 

Sig-1R plays a role in the pathogenesis of a variety of neurodegenerative diseases, including Alzheimer’s disease, Parkinson’s syndrome, and the lateral sclerosis of associated with muscular dystrophy [102]. Sig-1R is an important component of MAMs. A previous study showed that Sig-1R can be separated from Bip and combines with IP3Rs under conditions of mitochondrial stress, thus reducing the degradation of IP3Rs and promoting the transfer of Ca^2+^ to the mitochondria [103]. Caveolin-1 is known for that it helps to order the lipid bilayers organization [104]; moreover, it can directly interact with IP3Rs to promote the release of Ca^2+^ [105], while Ras can affect the subcellular distribution of Caveolin-1, thereby reducing the transfer of Ca^2+^ [106]. During the process of apoptosis, the tumor suppressor BRCA1 can be recruited to the surface of the ER in an IP3Rs-dependent manner and bind directly to IP3R1 to increase its sensitivity to IP3 and promote the transfer of Ca^2+^ to the mitochondria [107]. WFS1, NCS1, and IP3Rs, can form a complex to increase the connectivity between the ER and the mitochondria by enhancing Ca^2+^ transfer [108]. Research has shown that the mutation of WFS1 is responsible for a variety of abnormalities in the neurological and endocrine systems [109]. Tespa1 can be incorporated in MAMs and participate in the regulation of Ca^2+^ transfer by way of forming a complex with IP3Rs and Grp75. The knockdown of Tespa1 can down-regulate the level of Ca^2+^ in the mitochondria and cytoplasm [110,111]. ERO1α is also known to increase the activity of IP3Rs and promote apoptosis [112]. 

##### SERCAs

As the only channel responsible for the uptake of Ca^2+^ in the ER so far, SERCAs can also aggregate in MAMs. Furthermore, a variety of proteins in MAMs can regulate the function of SERCAs and affect the transfer of calcium signals. When incorporated in MAMs, P53 can change the redox state of SERCAs and promote its functional activity, thereby elevating the level of Ca^2+^ in the ER; this increases the flux of Ca^2+^ to the mitochondria, and promotes the occurrence of apoptosis [113,114]. TMX1 can negatively regulate the function of SERCA2b, and tumor cells with low expression levels of TMX1 exhibit higher levels of Ca^2+^ in the ER [115,116]. The anti-apoptotic protein Bcl-2 can directly bind to SERCAs to change its conformation, thereby down-regulating its functional activity and inhibiting the enrichment process of Ca^2+^ in the ER [117]. SEPN1 and ERO1α antagonize each other and work by regulating the redox state of SERCA2. SEPN1 can down-regulate the cysteine oxidation level of SERCA2 to maintain the stability of SERCA2 function [118].

In addition, 25 transcriptional variants of SERCA1 were detected in normal liver cells, among which 8 clones were found to be characterized by exon 11 splicing, named S1Ts. Further study revealed that S1Ts are expressed in different human tissues, such as adult pancreas, liver, kidney, lung, and placenta and fetal kidney, liver, brain, thymus [119]. S1Ts, which are localized in MAMs, can be induced by ER stress through PERK-eIF2α-ATF4 pathway and whose induction triggers Ca^2+^ leak from ER by forming homodimers in the ER membrane, which promotes the transfer of Ca^2+^ to mitochondria [119,120].

##### MCU

Although MCU and its regulatory proteins play crucial role in the uptake of Ca^2+^ by the mitochondria [79], there is no report showing specific accumulation of MCU at MAMs structure. However, MAMs can still influence the efficiency of MCU in Ca^2+^ uptake. Since the transport of Ca^2+^ by MCU is highly dependent on the “hot spot” effect of ER Ca^2+^ release, the MAMs proteins that can influence the spatial connection between the ER and the mitochondria (as detailed above) may influence the Ca^2+^ delivery efficiency by MCU indirectly (Table 1).

**Table 1:** Proteins that play roles in MAMs structure and function regulation.

Functions in MAMs	Proteins	Other Functions
	α-Synuclein	Unknown
	CypD	Regulation of MPTP [121]
	DJ-1	Antioxidant stress, chaperon activity, transcriptional regulation, degradation of proteins [122]
	FATE1	Tolerance to cellular stress [123]
	FUNDC1	Acts as mitophagy receptor [124]
Structure maintenance	NogoB	Regulation of ER morphology [69]
	PERK	Participating in ERS [125]
	PDK4	Regulation of cellular metabolism and mitochondrial function [126]
	Presenilin-2	Involved in cell adhesion, apoptosis and several cell-signaling processes [127]
	TDP-43 and FUS	RNA processing and transportation [128]
	TG2	Modification of proteins [63]
	TpMs	Unknown
	Akt	Regulation of cell growth and differentiation [129]
	Bcl2	Regulation of apoptosis [13]
	Bcl-xl	Regulation of apoptosis [13]
	Bok	Regulation of apoptosis and mitochondrial fusion/fission [130]
	BRCA1	A tumor suppressor, repair of DNA damage [107,131]
	Caveolin-1	Participating in the regulation of the cell cycle and cellular senescence, proliferation and invasion, cell death as well as membrane composition, lipid homeostasis and metabolism [132]
	ERO1α	Protein folding [133]
	MCL1	Regulation of apoptosis [13]
Regulating function of IP3Rs	mTORC2	Participating in multiple cellular processes such as proliferation, apoptosis, and differentiation [134]
	NCS1 and WFS1	Contributing to the maintenance of intracellular calcium homeostasis and regulation of calcium-dependent signaling pathways [135]; regulation of ER stress signaling [108]
	PML	Functions as a tumor suppressor and also involved in multiple cellular activities [136]
	PTEN	Tumor suppressor and metabolic regulator [137]
	Ras	Participating in proliferation, differentiation, apoptosis, senescence, and metabolism [138]
	Sig1-R	Regulation of ER stress, function of mitochondria, and oxidative stress, etc., [139]
	Tespa1	signaling molecule in thymocyte development [140]
	Bcl2	Regulation of apoptosis [13]
	CHOP	Participating in ER stress and apoptosis regulation [141]
Regulating function of SERCAs	ERO1α and SEPN1	Regulation of protein folding and secretion and inhibiting apoptosis, and regulates tumor progression [142]; regulation of oxidative stress [143]
	P53	Multiple roles in cellular activities [144]
	PMX1	Participating in protein folding [116,145]
	S1Ts	Variants of SERCA1 [119,120]

### 4.2. PACS2 Participates in the Transduction of Apoptosis Signals from the ER to the Mitochondria

PACS2 is a sorting protein located in the ER membrane which participates in the regulation of ER homeostasis, lipid synthesis, and the structural connection between the ER and the mitochondria. In addition, PACS2 mediates the process of apoptotic signal transduction from the ER to the mitochondria. High levels of ERS can cause translocation of the full-length Bid-bound PACS2 from the ER to the mitochondria; Bid is then cleaved on the surface of the mitochondria by caspase-8. Later, Bid interacts with Bax/Bak to promote permeability of the outer membrane of the mitochondria and the release of cytochrome c, thus initiating apoptosis [21].

### 4.3. The Role of LIPID Metabolism in the Transduction of Apoptosis Signals

MAMs plays a central role in lipid metabolism. A variety of lipids are synthesized in MAMs; some enzymes involved in the synthesis of triglyceride, ceramide, and sterol (fatty acid CoA ligase (ACS) 1/4) [146], acyl-coenzyme A: cholesterol acyltransferase-1 (ACAT1/SOAT1) [147] are known to exist only in MAMs [148]. Several lipid metabolites can affect cell fate. Of these, ceramide is the most typical. Under normal circumstances, ceramide is synthesized by the ceramide synthase pathway. However, under stress conditions (e.g., heat shock, TNF-α, Fas, chemotherapeutics, toxins, radiation and other factors) ceramide can be synthesized rapidly from sphingomyelin in the nerve sheath phospholipase [149]. The accumulation of ceramide can not only regulate and interact (directly or indirectly) with a variety of molecules involved in the transduction of apoptotic signals transduction, such as protein phosphatase 1A/2A, protein kinase C, and NF-κB, ras [150,151,152,153,154], a significant accumulation of ceramide can result in the formation of pores on the outer mitochondrial membrane, induce the release of pro-apoptotic substances, such as cytochrome c, in the intermembrane space of mitochondria [155,156], and transfer stress signals from the ER to the mitochondria. Inhibiting the activity of ACS1/4 can reduce the synthesis of ceramide and reduce the occurrence of apoptosis [157].

### 4.4. The Fis1-BAP31 Complex Is Involved in the Transduction of Apoptosis Signals

#### 4.4.1. The Relationship between Fis1 and Apoptosis

As the receptor for Drp1 during mitochondrial fission, Fis1 not only plays an important role in mitochondrial fission, but also participates in the process of apoptosis. First, the downregulation of Fis1 can reduce the release of cytochrome c in apoptotic cells and can inhibit the translocation of pro-apoptotic Bcl2 family members to the mitochondria [158]. Second, the overexpression of Fis1 can induce cell apoptosis via a Bax/Bak-independent process [159,160]. Although many studies have found that mitochondrial fission is often accompanied by the release of mitochondrial apoptosis-related substances, it is currently believed that when considering the relationship between mitochondrial fission and apoptosis, mitochondrial fission is the result of cell apoptosis rather than the cause. The results of multiple time-lapse microscopy experiments have confirmed that the release of cytochrome c from mitochondria when stimulated with apoptosis-inducing drugs occurs earlier than mitochondrial fission, and that the release of cytochrome c can also occur in both reticulated and tubular mitochondria [161,162]. Therefore, Fis1 is involved in the process of apoptosis and plays a specific role that occurs downstream of mitochondrial apoptosis.

#### 4.4.2. Fis1-BAP31 Participates in Apoptosis Signal Transduction from the Mitochondria to the ER

As a participant in the process of apoptosis, Fis1 can connect with BAP31 in MAMs to transmit mitochondrial apoptotic signals to the ER. BAP31 is an important chaperone protein on the ER membrane and is involved in the degradation of misfolded proteins and apoptosis within the ERS pathway. When Fis1 binds to BAP31, it can cleave BAP31 to produce p20 BAP31; as a pro-apoptotic protein, p20 BAP31 can convert procaspase-8 into an functional form that can truncate Bid and initiate apoptosis [21,163]. The activation of p20 BAP31 can also promote the transfer of Ca^2+^ from the ER to the mitochondria, thus showing that apoptosis signals can return back to the mitochondria [164,165], thus forming an amplification loop of apoptotic signals between the ER and the mitochondria that can help to coordinate the functions of these two organelles.

## 5. Methods of Detection

### 5.1. Fluorescence Microscopy

Fluorescence microscopy provides a preliminary solution for studying the structure of MAMs. Some researchers have used the co-localization of markers for the ER and mitochondria to study the connectivity between these two organelles [49,53]. However, in confocal fluorescence microscopy, the resolution of the *z*-axis is only 700 nm. Even though the most advanced microscopes can reach 300–400 nm [166], this is still not sufficient to measure the spatial gaps in MAMs (usually less than 100 nm). This means that results obtained via fluorescence microscopy may be questionable, although fluorescence microscopy still has certain advantages. First, fluorescence microscopy can observe living cells, making it possible to study the dynamic structure of MAMs [167]; second, with the application of a variety of fluorescent labels and proteins, the study of MAMs can be more targeted and efficient [38]. Consequently, fluorescence microscopy remains an indispensable method to study MAMs.

### 5.2. Transmission Electron Microscopy

Transmission electron microscopy is an irreplaceable tool for studying the structure of MAMs. The high-resolution imaging of transmission electron microscopy makes it possible to quantify the structure of MAMs [168]. At the same time, three-dimensional reconstruction technology after electron tomography can rebuild the structure of MAMs between the ER and mitochondria, making it possible to study the spatial morphology of MAMs [169,170,171]. This can provide a new perspective in the study of MAMs besides gap measurement and contact point quantification [144,172]. In addition, the combination of fluorescence microscopy and electron microscopy technology can be highly complementary and allow the study of both structure and function [159].

### 5.3. Gradient Centrifugation

Gradient centrifugation is a classic method for studying MAMs [173]. In combination with methods such as immunoblotting, immunofluorescence, mass spectrometry, and proteomics, the molecular composition of MAMs can be analyzed qualitatively and quantitatively [33]. In this process, gradient centrifugation is an important means to separate the structure of MAMs. However, there are also reports describing the use of biotin to separate and purify the structure and composition of MAMs and thus allow the qualitative and quantitative investigation of the composition of MAMs [174,175].

### 5.4. The Functional Evaluation of MAMs

The functional evaluation of MAMs can indirectly reflect the changes in the level of contact between the ER and the mitochondria. The synthesis of phospholipids and their transportation between the ER and the mitochondria, or the flux of Ca^2+^ transfer from the ER to the mitochondria, are usually used as indicators to reflect changes in the level of connectivity between the ER and the mitochondria [176,177].

## 6. Conclusions

As the most direct communicating medium between the ER and the mitochondria, MAMs play an important role in coordinating the multiple array of functions carried out by these two organelles, particularly the integration of apoptotic signals. The ER is the main site for intracellular protein modification, Ca^2+^ storage, and lipid synthesis. It is also evident that the ER is more susceptible to various stresses, and therefore plays a key role as a stress sensor and provides an initial response to stress. Mitochondria play an irreplaceable role in important cellular processes such as energy metabolism and apoptosis; consequently, it is logical that the mitochondria work downstream of the stress response. MAMs are located directly between these two organelles and are tightly involved in the transduction of stress signals from the ER to the mitochondria; in some cases, they transmit apoptotic signals back to the ER. This not only ensures the complementarity of the functions between the two organelles, but also amplifies apoptotic signals between the two organelles, thus promoting coordinated functional responses. Clarifying this process will provide a new perspective for the study of pathological mechanisms, such as tumors, neurodegenerative diseases, and aging.

Although some progress has been made in the study of MAMs over recent years, there are still some uncertainties that need to be addressed. First, in terms of structure, some proteins are directly involved in the maintenance of MAMs structure, while others work as regulators for structural maintenance. However, little is known about such proteins or how they can be delineated between the two organelles with respect to functionality. Second, in terms of function, especially in the regulation of Ca^2+^ transfer, the width of the gap between the ER and the mitochondria plays an important role. However, interfering SERCAs or IP3Rs can also affects the transfer of Ca^2+^. It is still not clear whether the proteins that can affect Ca^2+^ transfer in MAMs work by changing the tightness of the gap within MAMs or by disturbing the function of SERCA/IP3Rs. These unresolved issues will be an important direction for future research.

## Abbreviations

ACAT1/SOAT1	Acyl-Coenzyme A: Cholesterol Acyltransferase-1
ACS	Fatty acid CoA ligase
AD	Alzheimer’s disease
AIF	Apoptosis inducing factor
Akt	Serine/threonine-protein kinases
ALS	Amyotrophic lateral sclerosis
ATAD3A	ATPase family AAA domain-containing protein 3A
ATF4	Activating Transcription Factor 4
BAP31	B-cell receptor associated protein 31
Bip	Immunoglobulin heavy chain binding protein
BRCA1	Breast cancer susceptibility gene
CypD	Cyclophilin D
IRE1	Inositol-requiring enzyme 1
eIF2α	eukaryotic Initiation Factor 2α
ER	Endoplasmic reticulum
ERO1α	Endoplasmic reticulum oxidoreductase 1-α
ERS	Endoplasmic reticulum stress
ETC	Electron transport chain
FADD	Fas-associating protein with a novel death domain
FasL	Fas ligand
FATE1	Fetal and adult testis expressed 1
Fis1	Mitochondrial fission 1
FTD	Frontotemporal dementia
FUNDC1	FUN14 domain containing 1
FUS	Fused in sarcoma
Grp75	75 KDa Glucose-regulated protein
GSK-3b	Glycogen synthase kinase 3 beta
IP3Rs	Inositol1,4,5-trisphosphatereceptors
MAMs	Mitochondria associated membranes
MCU	Mitochondrial Ca(2+) uniporter
Mfn1/2	Mitofusins 1/2
MOSPD2	Motile sperm domain-containing protein 2
mPTP	Mitochondrial permeability transition pore
mTORC2	Mammalian target of rapamycin complex2
NCS1	Nucleobase cation symporter-1
NF-κB	Nuclear factor kappa B
PACS2	Phosphoacidic cluster sorting protein 2
PD	Parkinson’s disease
PDK4	Pyruvate dehydrogenase kinase 4
PERK	Protein kinase R (PKR)-like endoplasmic reticulum kinase
PTPIP51	Protein tyrosine phosphatase interacting protein 51
REEP1	Receptor expression-enhancing protein 1
ROS	Reactive oxygen species
RyRs	Ryanodine receptors
SEPN1	Selenoprotein N
SERCAs	Sarco(endo)plasmic reticulum calcium-ATPases
Sig-R	ECF sigma factor sigma(R)
S1Ts	Exon 4 and/or exon 11 spliced SERCA1 splice variants
TDP-43	TAR DNA binding protein 43
TG2	Transglutaminase 2
TMX1	Transmembrane thioredoxin-related protein 1
TNFR1	Tumor necrosis factor receptor 1
TpMs	Trichoplein keratin filament binding protein
TRADD	TNF receptor-associated death domain
VABP	Vitamin A binding protein
VDAC	Voltage-dependent anion channel
WASF3	Wiskott-Aldridge syndrome family 3
WFS1	Wolfram syndrome type 1
XBP1	X-box binding protein 1
